# Foods, nutrients and prostate cancer: a case–control study in Uruguay

**DOI:** 10.1038/sj.bjc.6690396

**Published:** 1999-05-01

**Authors:** H Deneo-Pellegrini, E De Stefani, A Ronco, M Mendilaharsu

**Affiliations:** Department of Pathology, Instituto Nacional de Oncología, Montevideo, Uruguay; Registro Nacional de Cáncer, Montevideo, Uruguay

## Abstract

A case–control study of diet and prostate cancer was conducted in Montevideo, Uruguay involving 175 cases and 233 controls. When the highest quartile of intake was compared with the lowest, positive findings were obtained for red meat intake (OR 2.0, 95% CI 1.1–3.8), desserts (OR 1.8, 95% CI 0.9–3.3), total energy (OR 1.9, 95% CI 1.0–3.4) and total fat intake (OR 1.8, 95% CI 0.9–3.4). On the other hand, vegetables and fruits (OR 0.5, 95% CI 0.3–0.9), vitamin C (OR 0.4, 95% 0.2–0.8) and vitamin E (OR 0.6, 95% CI 0.3–1.1) were associated with reduced risks of prostate cancer. Possible mechanisms are discussed. © 1999 Cancer Research Campaign


British Journal of Cancer (1999) 80(3/4), 591–597
© 1999 Cancer Research Campaign
Article no. bjoc.1998.0396

### Foods, nutrients and prostate cancer: a case–control study in Uruguay

**H Deneo-Pellegrini[1], E De Stefani[2], A Ronco[2] and M Mendilaharsu[2]**

[1]Department of Pathology, Instituto Nacional de Oncología, Montevideo, Uruguay; [2]Registro Nacional de Cáncer, Montevideo, Uruguay

**Summary** A case–control study of diet and prostate cancer was conducted in Montevideo, Uruguay involving 175 cases and 233 controls. When the highest quartile of intake was compared with the lowest, positive findings were obtained for red meat intake (OR 2.0, 95% CI 1.1–3.8), desserts (OR 1.8, 95% CI 0.9–3.3), total energy (OR 1.9, 95% CI 1.0–3.4) and total fat intake (OR 1.8, 95% CI 0.9–3.4). On the other hand, vegetables and fruits (OR 0.5, 95% CI 0.3–0.9), vitamin C (OR 0.4, 95% 0.2–0.8) and vitamin E (OR 0.6, 95% CI 0.3–1.1) were associated with reduced risks of prostate cancer. Possible mechanisms are discussed.

Prostate cancer is the second commonest malignancy among Uruguayan men, with an age-adjusted incidence rate of 32.6 per 100 000 (Parkin et al, 1997). According to a previous study (De Stefani et al, 1994), the mortality rate for prostate cancer has increased by 77% in the period between 1953 and 1991. Also, migrants from Spain and Italy have increased their risk of prostate cancer after arrival in Uruguay, suggesting the importance of environmental factors (De Stefani et al, 1990).

In the only previous analytic study conducted in Uruguay (De Stefani et al, 1995), diet was assessed by food groups; both red meat and dairy foods were associated with an increased risk of prostate cancer. Also, fruit intake was associated with a risk increase of 70% (De Stefani et al, 1995). Since these estimates were not energy-adjusted some uncertainty remains about its validity. Therefore, we have decided to carry out a new case–control study on dietary factors and prostate cancer, based on a more detailed food-frequency questionnaire.

### SUBJECTS AND METHODS

Selection of cases. In the period 1994–1997, all incident- and histologically verified prostatic adenocarcinomas occurring in men in the age range 40–89 years, admitted to the four major hospitals in Montevideo, were considered eligible for this study. Of 190 cases identified, 15 patients refused interview, leaving 175 cases of prostate carcinomas (response rate 92.1%). The stage distribution was as follows: localized 25%, regional 72% and disseminated 3%. There were no cases with latent carcinomas, and, therefore, this series is representative of a series of mainly advanced prostate tumours. The stage distribution of our series was compared with the figures drawn from the National Cancer Registry. According to this source, 70% of prostate cancers were locally advanced (regional) or disseminated at the time of the diagnosis. These figures reflect the fact that there are no mass screening programmes for prostate cancer in Uruguay.

*Received 27 May 1998*
*Revised 28 July 1998*
*Accepted 16 September 1998*

*Correspondence to:* E De Stefani, Director, Registro Nacional de Cáncer, Avda. Brasil 3080 dep. 402, Montevideo, Uruguay

### Controls selection

In the same period, all patients admitted to the same hospitals as the cases with conditions unrelated to diet were considered eligible as controls if below age 90. A total of 240 patients were hospital-matched to the cases; from this initial number seven patients refused interview, leaving a total of 233 controls (response rate 97.1%). The distribution of controls by disease category was as follows: eye disorders (87 patients, 37.3%), abdominal hernia (56 patients, 24.0%), acute appendicitis (25 patients, 10.7%), fractures and trauma (23 patients, 9.9%), hydatid cyst (15 patients, 6.4%), skin diseases (14 patients, 6.1%) and varicose veins (13 patients, 5.6%).

### Questionnaire

Both cases and controls were specifically called up to the hospital for a face-to-face interview after diagnosis or treatment. The mean time since admission for cases was 62 days, and for controls was 50 days. Both cases and controls completed a detailed questionnaire which covered sociodemographic variables, anthropometric variables, occupational exposures, family history of cancer, tobacco history, alcohol consumption and diet. The food-frequency questionnaire included 64 food items, representative of usual diet of the Uruguayan population. This food-frequency questionnaire was not previously validated but was studied regarding its reproducibility. The Pearson correlation coefficients ranged from 0.30 for calcium to 0.79 for total carbohydrate intake. For each food, a commonly used unit or portion size was specified, and participants were asked how often, on average, over the past year or the year prior to onset of symptoms, they had consumed that amount of each food. The responses were open-ended allowing each food to be treated as a continuous variable (Willett, 1990). Responses were converted to times per year, multiplying by the appropriate time units. We consider that this type of recording food consumption reflects the true consumption more accurately, instead of forcing responses into pre-existing categories. The following food groups were analysed in this study:

1. Red meat, i.e. beef and lamb
2. White meat, i.e. poultry and fish
3. Processed meat, i.e. sausage, bacon, salami, saucisson, mortadella, ham and salted meat
4. Offal, i.e. tripe, kidney and liver
5. Total meat, i.e. the sum of the previous items
6. Dairy foods, i.e. cheese, butter, whole milk and ice cream
7. Desserts, i.e. rice pudding, custard, cake, marmalade and jam
8. Eggs, i.e. poached, boiled and fried eggs
9. Grains, i.e. rice, polenta, pasta, bread and croissants
10. Tubers, i.e. potato and sweet potato
11. Legumes, i.e. kidney beans and lentils
12. Vegetables and fruits, i.e. carrot, tomato, lettuce, onion, garlic, swiss chard, spinach, cabbage, cauliflower, winter squash, zucchini, red pepper, orange, orange juice, apple, peach, pear, grapes, figs, banana and fruit cocktail.

Nutrient indices were derived from local food tables (Mazzei and Puchulu, 1996). Since values for beta-carotene and other carotenoids are not available in Uruguay, the estimates of Mangels et al were used (1993).

**Table 1** Distribution of cases and controls by selected variables

| Variable: Category | Cases | Controls |
|---|---|---|
| Hospital | | |
| Cancer Institute | 50 (28.6) | 65 (27.9) |
| Pasteur | 29 (16.6) | 38 (16.3) |
| University | 77 (44.0) | 105 (45.1) |
| Maciel | 19 (10.8) | 25 (10.7) |
| Age (years) | | |
| 40–49 | 2 (1.1) | 3 (1.3) |
| 50–59 | 7 (4.0) | 22 (9.4) |
| 60–69 | 54 (30.9) | 83 (35.6) |
| 70–79 | 87 (49.5) | 103 (44.2) |
| 80–89 | 25 (14.3) | 22 (9.4) |
| Residence | | |
| Montevideo | 85 (48.6) | 122 (52.4) |
| Other counties | 90 (51.4) | 111 (47.6) |
| Urban/rural status | | |
| Urban | 118 (67.4) | 178 (76.4) |
| Rural | 57 (32.6) | 55 (23.6) |
| Education (years) | | |
| 0–2 | 58 (33.1) | 70 (30.0) |
| 3–5 | 67 (38.3) | 73 (31.3) |
| 6+ | 50 (28.6) | 90 (38.6) |
| Monthly income (US dollars) | | |
| <157 | 48 (27.4) | 73 (31.3) |
| 158+ | 49 (28.0) | 69 (29.6) |
| Unknown | 78 (44.6) | 91 (39.1) |
| Family history of prostate cancer | | |
| No | 168 (96.0) | 232 (99.6) |
| Yes | 7 (4.0) | 1 (0.4) |
| Number of patients | 175 (100) | 233 (10) |

### Statistical analysis

Crude and adjusted odds ratios (OR) and the corresponding 95% confidence intervals (CI) were calculated by multiple logistic regression (Breslow and Day, 1980). In all models, potential confounders were included. These were: age (continuous), residence (Montevideo vs other counties), urban/rural status (urban vs rural), family history of prostate cancer (no vs yes), body mass index (continuous) and total energy intake (continuous). Since tobacco consumption and alcohol intake were not associated with prostate cancer risk in this dataset, they were not included in the logistic models. Odds ratios for food groups were calculated with and without a term for total energy intake. All food groups and nutrients were tested for interaction with the following variables: total energy intake, body mass index, and age dichotomized in younger than 70 years and 70 years or more. Energy intake and body mass index were dichotomized according to the median value of the combined sample of cases and controls. Nutrients were energy-adjusted by the residuals method (Willett and Stampfer, 1986).

The test for trend after multivariate adjustment for covariates was determined by the $\chi^2$ statistic across the vector of indicator variables for the exposure of interest. All calculations were performed in the GLIM program (Baker and Nelder, 1985).

### RESULTS

Sociodemographic variables and family history of prostate cancer in a first-degree relative are shown in Table 1. Cases were older, lived more frequently outside Montevideo, were more frequently rural residents and were less educated than controls. Although these differences were not statistically significant, the above mentioned variables were included in all following logistic models, in order to control confounding. On the other hand, family history of prostate cancer was much more frequent among cases than controls (crude OR 9.8).

Odds ratios of prostate cancer for food groups are shown in Table 2. In the models without a term for total energy intake, intake in the uppermost quartile compared with the bottom quartile for red meat, total meat, desserts, grains and tubers displayed increased risks (OR for red meat 2.0, 95% CI 1.1–3.8). However, reduced risks were observed for the following groups: all vegetables, all fruits and all vegetables and fruits (OR for all vegetables and fruits in the uppermost quartile of intake compared with the lower quartile was of 0.6, 95% CI 0.3–1.1). When total energy intake was introduced in the model, red meat intake was no longer significant (OR 1.7, 95% CI 0.8–3.4, *P*-value for trend 0.17). Desserts intake was associated with a moderate increased risk (OR 1.8, 95% CI 0.9–3.3), whereas vegetables, and vegetables and fruits together, were associated with reduced risks (OR for all vegetables and fruits 0.5, 95% CI 0.3–0.9, *P*-value for linear trend 0.04).

Odds ratios of prostate cancer for nutrients are shown in Table 3. Whereas protein was not associated with risk, carbohydrate intake was associated with a reduced risk of 0.5 (95% CI 0.3–1.0). Total fat displayed an increased risk of 1.8 for the uppermost quartile of intake (95% CI 0.8–3.4). Saturated fat was not associated with risk, and cholesterol intake displayed an increased risk of 2.4 (95% CI 1.3–4.4) in the third quartile, which decreased to zero in the highest quartile of intake. Among carotenoids, only lutein

© Cancer Research Campaign 1999

**Table 2** Odds ratios of prostate cancer for food groups

| **Food group** | **Quartile** I | II | III | IV | ***P* for trend** |
|---|---|---|---|---|---|
| Red meat | | | | | |
| IQR[c] | ≤182 | 183–365 | 366–378 | 379+ | |
| Cases/Controls | 32/71 | 61/78 | 36/40 | 46/44 | |
| OR1[a] | 1.0 | 1.6 | 1.8 | 2.0 | |
| 95% CI | – | 0.9–2.9 | 0.9–3.5 | 1.1–3.8 | 0.03 |
| OR2[b] | 1.0 | 1.5 | 1.7 | 1.7 | |
| 95% CI | – | 0.9–2.7 | 0.9–3.3 | 0.8–3.4 | 0.17 |
| White meat | | | | | |
| IQR | ≤24 | 25–64 | 65–104 | 105+ | |
| Cases/Controls | 44/58 | 41/65 | 51/59 | 39/51 | |
| OR1 | 1.0 | 0.9 | 1.2 | 1.1 | |
| 95% CI | – | 0.5–1.5 | 0.7–2.1 | 0.6–2.0 | 0.51 |
| OR2 | 1.0 | 0.8 | 1.1 | 0.9 | |
| 95% CI | – | 0.5–1.5 | 0.6–1.9 | 0.5–1.8 | 0.86 |
| Poultry | | | | | |
| IQR | ≤12 | 13–40 | 41–52 | 53+ | |
| Cases/Controls | 45/75 | 26/32 | 64/83 | 40/43 | |
| OR1 | 1.0 | 1.3 | 1.3 | 1.5 | |
| 95% CI | – | 0.7–2.6 | 0.8–2.2 | 0.8–2.6 | 0.18 |
| OR2 | 1.0 | 1.3 | 1.2 | 1.3 | |
| 95% CI | – | 0.7–2.5 | 0.7–2.0 | 0.7–2.4 | 0.38 |
| Fish | | | | | |
| IQR | 0 | 1–18 | 19–52 | 53+ | |
| Cases/Controls | 41/61 | 50/59 | 60/73 | 24/70 | |
| OR1 | 1.0 | 1.3 | 1.4 | 0.9 | |
| 95% CI | – | 0.7–2.2 | 0.8–2.4 | 0.5–1.9 | 0.78 |
| OR2 | 1.0 | 1.3 | 1.3 | 0.9 | |
| 95% CI | – | 0.7–2.3 | 0.7–2.2 | 0.5–1.8 | 0.99 |
| Processed meat | | | | | |
| IQR | ≤182 | 183–365 | 366–378 | 379+ | |
| Cases/Controls | 41/60 | 48/56 | 46/54 | 40/63 | |
| OR1 | 1.0 | 1.2 | 1.1 | 0.9 | |
| 95% CI | – | 0.7–2.1 | 0.6–2.0 | 0.5–1.7 | 0.75 |
| OR2 | 1.0 | 1.2 | 1.0 | 0.8 | |
| 95% CI | – | 0.7–2.2 | 0.6–1.8 | 0.4–1.4 | 0.31 |
| Offal | | | | | |
| IQR | 0 | 1–24 | 25–52 | 53+ | |
| Cases/Controls | 74/119 | 36/49 | 33/25 | 32/40 | |
| OR1 | 1.0 | 1.2 | 2.1 | 1.2 | |
| 95% CI | – | 0.7–2.0 | 1.1–3.9 | 0.7–2.1 | 0.18 |
| OR2 | 1.0 | 1.3 | 2.1 | 1.1 | |
| 95% CI | – | 0.8–2.3 | 1.1–3.8 | 0.6–1.9 | 0.30 |
| Total meat | | | | | |
| IQR | ≤422 | 423–570 | 571–767 | 768+ | |
| Cases/Controls | 30/72 | 53/50 | 46/58 | 46/53 | |
| OR1 | 1.0 | 2.6 | 1.7 | 1.9 | |
| 95% CI | – | 1.4–4.7 | 0.9–3.1 | 1.1–3.6 | 0.11 |
| OR2 | 1.0 | 2.3 | 1.5 | 1.6 | |
| 95% CI | – | 1.3–4.4 | 0.8–2.9 | 0.8–3.4 | 0.59 |
| Dairy foods | | | | | |
| IQR | ≤312 | 313–469 | 470–729 | 730+ | |
| Cases/Controls | 43/61 | 46/56 | 40/56 | 46/60 | |
| OR1 | 1.0 | 1.1 | 1.1 | 1.1 | |
| 95% CI | – | 0.6–1.8 | 0.6–2.0 | 0.6–1.9 | 0.65 |
| OR2 | 1.0 | 0.9 | 0.9 | 0.8 | |
| 95% CI | – | 0.5–1.7 | 0.5–1.7 | 0.4–1.6 | 0.60 |
| Eggs | | | | | |
| IQR | ≤48 | 49–103 | 104–142 | 143+ | |
| Cases/Controls | 35/63 | 44/54 | 47/61 | 49/55 | |
| OR1 | 1.0 | 1.5 | 1.5 | 1.6 | |
| 95% CI | – | 0.8–2.7 | 0.8–2.6 | 0.9–2.9 | 0.12 |
| OR2 | 1.0 | 1.4 | 1.3 | 1.4 | |
| 95% CI | – | 0.8–2.5 | 0.7–2.4 | 0.7–2.6 | 0.41 |

*© Cancer Research Campaign 1999*

**Table 2** (Cont) Odds ratios of prostate cancer for food groups

| Food group | Quartile I | II | III | IV | *P* for trend |
|---|---|---|---|---|---|
| Desserts | | | | | |
| IQR | ≤24 | 25–90 | 91–182 | 183+ | |
| Cases/Controls | 41/65 | 40/62 | 41/62 | 53/44 | |
| OR1 | 1.0 | 1.0 | 1.1 | 2.0 | |
| 95% CI | – | 0.6–1.8 | 0.6–1.9 | 1.1–3.6 | 0.02 |
| OR2 | 1.0 | 1.0 | 1.0 | 1.8 | |
| 95% CI | – | 0.6–1.8 | 0.6–1.8 | 0.9–3.3 | 0.07 |
| Grains | | | | | |
| IQR | ≤ 812 | 813–924 | 925–1228 | 1229+ | |
| Cases/Controls | 38/65 | 51/58 | 33/61 | 53/49 | |
| OR1 | 1.0 | 1.5 | 1.0 | 2.1 | |
| 95% CI | – | 0.8–2.6 | 0.5–1.8 | 1.2–3.7 | 0.05 |
| OR2 | 1.0 | 1.5 | 0.9 | 1.7 | |
| 95% CI | – | 0.9–2.7 | 0.5–1.7 | 0.9–3.2 | 0.24 |
| Vegetables | | | | | |
| IQR | ≤ 336 | 337–466 | 467–696 | 697+ | |
| Cases/Controls | 48/55 | 52/52 | 36/63 | 39/63 | |
| OR1 | 1.0 | 1.3 | 0.7 | 0.7 | |
| 95% CI | – | 0.7–2.3 | 0.4–1.2 | 0.4–1.3 | 0.11 |
| OR2 | 1.0 | 1.2 | 0.6 | 0.6 | |
| 95% CI | – | 0.7–2.2 | 0.3–1.1 | 0.3–1.1 | 0.02 |
| Fruits | | | | | |
| IQR | ≤ 270 | 271–429 | 430–735 | 736+ | |
| Cases/Controls | 46/57 | 54/47 | 35/67 | 40/62 | |
| OR1 | 1.0 | 1.5 | 0.7 | 0.8 | |
| 95% CI | – | 0.8–2.6 | 0.4–1.2 | 0.5–1.6 | 0.21 |
| OR2 | 1.0 | 1.5 | 0.6 | 0.8 | |
| 95% CI | – | 0.8–2.7 | 0.3–1.1 | 0.4–1.4 | 0.08 |
| Vegetables and fruits | | | | | |
| IQR | ≤ 685 | 686–1000 | 1001–1389 | 1390+ | |
| Cases/Controls | 55/47 | 40/63 | 38/63 | 42/60 | |
| OR1 | 1.0 | 0.5 | 0.5 | 0.6 | |
| 95% CI | – | 0.3–0.9 | 0.3–0.9 | 0.3–1.1 | 0.13 |
| OR2 | 1.0 | 0.5 | 0.5 | 0.5 | |
| 95% CI | – | 0.3–0.8 | 0.3–0.8 | 0.3–0.9 | 0.04 |
| Legumes | | | | | |
| IQR | ≤ 6 | 7–24 | 25–52 | 53+ | |
| Cases/Controls | 45/68 | 70/80 | 18/31 | 42/54 | |
| OR1 | 1.0 | 1.4 | 0.9 | 1.2 | |
| 95% CI | – | 0.8–2.2 | 0.4–1.9 | 0.7–2.1 | 0.88 |
| OR2 | 1.0 | 1.4 | 0.8 | 1.1 | |
| 95% CI | – | 0.8–2.3 | 0.4–1.7 | 0.6–1.9 | 0.85 |
| Tubers | | | | | |
| IQR | ≤ 168 | 169–364 | 365–443 | 444+ | |
| Cases/Controls | 36/70 | 48/58 | 54/56 | 42/49 | |
| OR1 | 1.0 | 1.9 | 2.1 | 1.9 | |
| 95% CI | – | 1.0–3.4 | 1.1–3.7 | 1.0–3.5 | 0.04 |
| OR2 | 1.0 | 1.7 | 1.9 | 1.6 | |
| 95% CI | – | 0.9–3.2 | 1.0–3.4 | 0.8–3.1 | 0.17 |

[a]OR1 – Adjusted for age, residence, urban/rural status, education, family history of prostate cancer in a first-degree relative and body mass index. [b]OR2 – Further adjusted for total energy intake. [c]IQR – Interquartile Range in Servings.

displayed a moderate decreased risk of 0.70 (95% CI 0.4–1.3), but without a significant trend. Both vitamins C and E were associated with a protective effect (OR for vitamin C 0.4, 95% CI 0.2–0.8), and vitamin D displayed a moderate reduced risk of 0.7 (95% CI 0.4–1.2).

Odds ratios of prostate cancer for total fat and carbohydrate intakes by levels of body mass index are shown in Table 4. Whereas total fat intake was not associated with risk at low levels of body mass, a strong effect of fat was observed among more obese patients. On the other hand, carbohydrate intake at high body mass was associated with a reduction in risk of 70% (95% CI 0.1–0.7).

### DISCUSSION

The present study showed increased risks of prostate cancer associated with total energy, total fat, red meat and dessert intakes. The risk associated with fat intake was more evident among obese

*© Cancer Research Campaign 1999*

**Table 3** Odds ratios of prostate cancer for nutrients[a]

| | Quartile | | | | |
|---|---|---|---|---|---|
| **Nutrient** | **I** | **II** | **III** | **IV** | ***P* for trend** |
| Total energy | | | | | |
| IQR[b] | ≤ 1527 | 1528–1914 | 1915–2326 | 2327+ | |
| Cases/Controls | 38/66 | 40/59 | 46/57 | 51/51 | |
| OR | 1.0 | 1.2 | 1.5 | 1.9 | |
| 95% CI | – | 0.7–2.2 | 0.9–2.7 | 1.0–3.4 | 0.03 |
| Protein | | | | | |
| IQR[c] | ≤ 62.9 | 63.0–76.9 | 77.0–97.4 | 97.5+ | |
| Cases/Controls | 38/64 | 43/59 | 53/49 | 41/61 | |
| OR | 1.0 | 1.1 | 1.7 | 1.0 | |
| 95% CI | – | 0.6–1.9 | 0.9–3.1 | 0.6–1.8 | 0.60 |
| Carbohydrate | | | | | |
| IQR[c] | ≤ 188.5 | 188.5–244.3 | 244.4–301.7 | 301.8+ | |
| Cases/Controls | 48/54 | 48/54 | 48/54 | 31/71 | |
| OR | 1.0 | 1.0 | 1.1 | 0.5 | |
| 95% CI | – | 0.6–1.8 | 0.6–2.0 | 0.3–1.0 | 0.13 |
| Total fat | | | | | |
| IQR[c] | ≤ 53.7 | 53.8–66.7 | 66.8–82.2 | 82.3+ | |
| Cases/Controls | 34/68 | 46/56 | 49/53 | 46/56 | |
| OR | 1.0 | 1.6 | 2.0 | 1.8 | |
| 95% CI | – | 0.9–2.9 | 1.1–3.7 | 0.9–3.4 | 0.04 |
| Saturated fat | | | | | |
| IQR[c] | ≤ 20.1 | 20.2–25.8 | 25.9–32.7 | 32.8+ | |
| Cases/Controls | 39/63 | 49/53 | 46/56 | 41/61 | |
| OR | 1.0 | 1.4 | 1.2 | 0.9 | |
| 95% CI | – | 0.8–2.4 | 0.7–2.1 | 0.5–1.7 | 0.78 |
| Cholesterol | | | | | |
| IQR[d] | ≤ 288.9 | 289.0–398.7 | 398.8–522.6 | 522.7+ | |
| Cases/Controls | 32/70 | 40/62 | 55/47 | 48/54 | |
| OR | 1.0 | 1.8 | 2.4 | 1.0 | |
| 95% CI | – | 1.0–3.2 | 1.3–4.4 | 0.6–1.9 | 0.72 |
| Vitamin A | | | | | |
| IQR[e] | ≤ 6204 | 6205–9460 | 9461–15838 | 15839+ | |
| Cases/Controls | 43/59 | 49/53 | 44/58 | 39/63 | |
| OR | 1.0 | 1.3 | 1.0 | 0.8 | |
| 95% CI | – | 0.8–2.4 | 0.6–1.9 | 0.4–1.4 | 0.34 |
| Dietary fibre | | | | | |
| IQR[c] | ≤ 18.2 | 18.3–21.7 | 21.8–27.1 | 27.2+ | |
| Cases/Controls | 36/66 | 48/54 | 47/55 | 44/58 | |
| OR | 1.0 | 1.6 | 1.7 | 1.5 | |
| 95% CI | – | 0.9–2.9 | 0.9–3.2 | 0.8–2.6 | 0.18 |
| Sucrose | | | | | |
| IQR[c] | ≤ 12.1 | 12.2–19.2 | 19.3–29.6 | 29.7+ | |
| Cases/Controls | 39/63 | 47/55 | 45/57 | 44/58 | |
| OR | 1.0 | 1.2 | 1.4 | 1.0 | |
| 95% CI | – | 0.6–2.1 | 0.8–2.6 | 0.6–1.8 | 0.49 |
| Beta-carotene | | | | | |
| IQR[f] | ≤ 2705 | 2706–4270 | 4271–7484 | 7485+ | |
| Cases/Controls | 41/61 | 44/58 | 48/54 | 42/60 | |
| OR | 1.0 | 1.2 | 1.4 | 1.0 | |
| 95% CI | – | 0.6–2.1 | 0.8–2.6 | 0.6–1.8 | 0.79 |
| Alpha-carotene | | | | | |
| IQR[f] | ≤ 109 | 110–291 | 292–600 | 601+ | |
| Cases/Controls | 41/61 | 55/47 | 42/60 | 37/65 | |
| OR | 1.0 | 1.8 | 1.1 | 0.9 | |
| 95% CI | – | 1.0–3.3 | 0.6–1.9 | 0.5–1.6 | 0.40 |
| Lycopene | | | | | |
| IQR[f] | ≤ 1300 | 1301–2501 | 2502–3300 | 3301+ | |
| Cases/Controls | 41/61 | 51/51 | 36/66 | 47/55 | |
| OR | 1.0 | 1.6 | 0.8 | 1.2 | |
| 95% CI | – | 0.9–2.8 | 0.4–1.4 | 0.7–2.2 | 0.90 |

*© Cancer Research Campaign 1999*

**Table 3** (Cont) Odds ratios of prostate cancer for nutrients[a]

| Nutrient | Quartile I | II | III | IV | *P* for trend |
|---|---|---|---|---|---|
| Lutein | | | | | |
| IQR[f] | ≤ 1214 | 1215–2086 | 2087–3593 | 3594+ | |
| Cases/Controls | 44/58 | 51/51 | 43/59 | 37/65 | |
| OR | 1.0 | 1.3 | 0.9 | 0.7 | |
| 95% CI | – | 0.7–2.3 | 0.5–1.7 | 0.4–1.3 | 0.15 |
| Vitamin C | | | | | |
| IQR[d] | ≤ 85.8 | 85.9–115.6 | 115.7–161.8 | 161.9+ | |
| Cases/Controls | 55/47 | 45/57 | 39/63 | 36/66 | |
| OR | 1.0 | 0.6 | 0.5 | 0.4 | |
| 95% CI | – | 0.3–1.0 | 0.3–0.8 | 0.2–0.8 | 0.008 |
| Vitamin E | | | | | |
| IQR[d] | ≤ 5.0 | 5.1–6.0 | 6.1–7.8 | 7.9+ | |
| Cases/Controls | 49/53 | 52/50 | 37/65 | 37/65 | |
| OR | 1.0 | 1.2 | 0.6 | 0.6 | |
| 95% CI | – | 0.7–2.1 | 0.3–1.1 | 0.3–1.1 | 0.03 |
| Vitamin D | | | | | |
| IQR[e] | ≤ 75.2 | 75.3–148.4 | 184.5–189.7 | 189.8+ | |
| Cases/Controls | 44/58 | 52/49 | 46/57 | 33/69 | |
| OR | 1.0 | 1.3 | 0.9 | 0.7 | |
| 95% CI | – | 0.7–2.5 | 0.5–1.8 | 0.4–1.2 | 0.14 |

[a]Adjusted for age, residence, urban/rural status, education, family history of prostate cancer, body mass index and total energy intake. [b]Kcal per day; [c]Grams per day; [d]Miligrams per day; [e]IU; [f]Micrograms per day.

**Table 4** Odds ratios of prostate cancer for fat and carbohydrate intakes according levels of body mass index[a]

| | Body mass index | |
|---|---|---|
| *Fat* | *Low* | *High* |
| Low | 1.0 | 1.0 |
| 2 | 1.5 (0.7–3.1) | 2.3 (0.8–6.1) |
| 3 | 0.9 (0.5–2.1) | 4.2 (1.6–11.4) |
| High | 0.9 (0.4–1.9) | 3.3 (1.2–8.9) |
| *Carbohydrate* | *Low* | *High* |
| Low | 1.0 | 1.0 |
| 2 | 1.0 (0.4–2.3) | 0.9 (0.4–2.0) |
| 3 | 1.7 (0.8–3.6) | 0.6 (0.2–1.4) |
| High | 0.8 (0.4–1.8) | 0.3 (0.1–0.7) |

[a]Adjusted for age, residence, urban/rural status and total energy intake.

patients, and also after controlling for dietary fibre and vitamin E intakes (results not shown). As in previous studies (West et al, 1991; Rohan et al, 1995; Whittemore et al, 1995; Meyer et al, 1997), total energy intake was a risk factor for prostate cancer. Although it is difficult to disentangle the effects of total energy intake from the effects of energy-dense foods, the evidence suggests that high energy intake may increase the risk of prostate cancer (World Cancer Research Fund, 1997).

Previous studies and reviews have reported increased risks of prostate cancer associated with red meat intake (Kolonel and Nomura, 1992; Talamini et al, 1992; Boyle and Zaridze, 1993; Giovannucci et al, 1993; Pienta and Esper, 1993; Gann et al, 1994; Le Marchand et al, 1994). The mechanisms of red meat intake as a risk factor for this cancer site are mostly unknown, although red meat's fat content may be a factor, possibly immediated by androgenic hormones (World Cancer Research Fund, 1997). On the other hand, fried or broiled meat may be a source of heterocyclic amines, potent mutagens in experimental studies (Weisburger et al, 1994). These chemicals have proved to be associated with cancers at other sites (De Stefani et al, 1997) and further studies on prostate cancer and heterocyclic amine intake are needed. Total fat intake was associated with risk in our study as in previous studies (West et al, 1991; Giovannucci et al, 1993; Whittemore et al, 1995).

Both vitamin A and carotenoids have been the subject of conflicting reports (Boyle and Zaridze, 1993; Pienta and Esper, 1993; Giovannucci, 1995; Kolonel et al, 1987, 1988; Le Marchand et al, 1991) and, in our study, no clear association with either was found. On the other hand, vitamin C was associated with a rather strong protective effect. Previous studies on vitamin C intake and prostate cancer (West et al, 1991; Rohan et al, 1995) reported no significant association. Also vitamin E was associated with a reduced risk of prostate cancer in our study. A recent report from a clinical trial (Heinonen et al, 1998) showed a 32% decrease in prostate cancer, associated with supplementation with α-tocopherol. It has been suggested that this protective effect is related to the antioxidant effect of vitamin E. Thus, the protective effect of vegetables and fruits, vitamin C and vitamin E could be due to a mechanism against oxidative stress (Heinonen et al, 1998).

As in all hospital-based case-control studies, the present study has a number of limitations and strengths. Perhaps the major limitation is related to changes in the diet of control patients. Although studies on diseases accepted as control diseases have shown no major differences among the general population regarding intake of meat, vegetables, fruits and legumes, it is not possible to rule out the possibility of mis-classification bias, generally towards the null. Another limitation is related to the limited number of cases afflicted with prostate cancer, which precludes against strong statements in the consideration of the results. Among the strengths, the high response rate, both in cases and controls, reassures against selection bias.

*© Cancer Research Campaign 1999*

In summary, this case-control study suggests an increased risk of prostate cancer associated with total energy, total fat, red meat intake and a protective effect of vegetables, fruits, vitamin C and vitamin E intakes.

### REFERENCES

Baker RJ and Nelder JA (1985) *The GLIM System: release 3.77*. Numerical Algorithms Group: Oxford

Boyle P and Zaridze DG (1993) Risk factors for prostate and testicular cancer. *Eur J Cancer* **29A**: 1048–1055

Breslow NE and Day NE (1980) *Statistical Methods in Cancer Research*, Vol. I. *The Analysis of Case-control Studies*. IARC Scientific Publication No 32. IARC: Lyon

De Stefani E, Parkin DM, Khlat M, Vassallo A and Abella M (1990) Cancer in migrants to Uruguay. *Int J Cancer* **46**: 232–237

De Stefani E, Fierro L, Barrios E and Ronco A (1994) Cancer mortality trends in Uruguay 1953–1991. *Int J Cancer* **56**: 634–639

De Stefani E, Fierro L, Barrios E and Ronco A (1995) Tobacco, alcohol, diet and risk of prostate cancer. *Tumori* **81**: 315–320

De Stefani E, Ronco A, Mendilaharsu M, Guidobono M and Deneo-Pellegrini H (1997) Meat intake, heteroyclic amines, and risk of breast cancer. *Cancer Epidemiol Biomarkers Prev* **6**: 573–582

Gann PH, Hennekens CH, Sacks FM, Grodstein F, Giovannucci EL and Stampfer MJ (1994) Prospective study of plasma fatty acids and risk of prostate cancer. *J Natl Cancer Inst* **86**: 281–286

Giovannucci E (1995) Epidemiologic characteristics of prostate cancer. *Cancer* **75**: 1766–1777

Giovannucci E, Rimm EB, Colditz GA, Stampfer MJ, Ascherio A, Chute CC and Willett WC (1993) A prospective study of dietary fat and risk of prostate cancer. *J Natl Cancer Inst* **85**: 1571–1579

Heinonen OP, Albanes D, Virtamo J, Taylor PR, Hattunen JK, Hartman AM, Haapakoski J, Malila N, Rautalahti M, Ripatti S, Mäenpää H, Teerenhovi L, Koss L, Virolainen M and Edwards BK (1998) Prostate cancer and supplementation with alpha-tocopherol and beta-carotene: incidence and mortality in a controlled trial. *J Natl Cancer Inst* **90**: 440–446

Kolonel LN, Hankin JH and Yoshizawa CN (1987) Vitamin A and prostate cancer in elderly men: enhancement of risk. *Cancer Res* **47**: 2982–2985

Kolonel LN, Yoshizawa CN and Hankin JH (1988) Diet and prostatic cancer: a case-control study in Hawaii. *Am J Epidemiol* **127**: 999–1012

Kolonel LN and Nomura AM (1992) Dietary intervention trials on prostate cancer. In *Macronutrients*: *Investigating Their Role in Cancer*, Micozzi M and Moon T (eds), pp. 423–436. Marcel Dekker: New York

Le Marchand L, Hankin JH, Kolonel LN and Wilkens LR (1991) Vegetable and fruit consumption in relation to prostate cancer risk in Hawaii: a re-evaluation of the effect of dietary beta-carotene. *Am J Epidemiol* **133**: 215–219

Le Marchand L, Kolonel LN and Wilkens LR (1994) Animal fat consumption and prostate cancer: a prospective study in Hawaii. *Epidemiology* **5**: 276–282

Mangels AR, Holden JM, Beecher GR, Forman MR and Lanza E (1993) Carotenoid content of fruits and vegetables: an evaluation of analytic data. *J Am Diet Assoc* **93**: 284–296

Mazzei ME and Puchulu MR (1991) *Table of Chemical Composition of Foods*. Cenexa: Buenos Aires (in Spanish)

Meyer F, Bairati I, Fradet Y and Moore L (1997) Dietary energy and nutrients in relation to preclinical prostate cancer. *Nutr Cancer* **29**: 120–126

Parkin DM, Whelan SL, Ferlay J, Raymond L and Young J (1997) *Cancer Incidence in Five Continents*, Vol. VII. IARC Scientific Publication No 143. IARC: Lyon

Pienta KJ and Esper PS (1993) Risk factors for prostate cancer. *Ann Intern Med* **118**: 793–803

Rohan TE, Howe GR, Burch JD and Jain M (1995) Dietary factors and risk of prostate cancer. *Cancer Causes Control* **6**: 145–154

Talamini R, Franceschi S, La Vecchia C, Serraino D, Barra S and Negri E (1992) Diet and prostatic cancer: a case-control study in Northern Italy. *Nutr Cancer* **18**: 277–286

Weisburger JH, Rivenson A, Hard GC, Zang E, Nagao M and Sugimura T (1994) Role of fat and calcium in cancer causation by food mutagens, heterocyclic amines. *Proc Soc Exp Biol Med* **205**: 347–352

West DW, Slattery ML, Robison LM, French TK and Mahoney AW (1991) Adult dietary intake and prostate cancer risk in Utah: a case–control study with special emphasis on aggressive tumors. *Cancer Causes Control* **2**: 85–94

Whittemore AS, Kolonel LN, Wu AH, John EM, Gallagher RP, Howe GR, Burch JD, Hankin J, Dreon DM, West DW, Teh C-Z and Paffenbarger RS (1995) Prostate cancer in relation to diet, physical activity, and body size in blacks, whites, and Asians in the United States and Canada. *J Natl Cancer Inst* **87**: 652–661

Willett WC (1990) *Nutritional Epidemiology*. Oxford University Press: New York

Willett WC and Stampfer MJ (1986) Total energy intake: implications for epidemiologic analyses. *Am J Epidemiol* **124**: 17–27

World Cancer Research Fund (1997) *Food, Nutrition and the Prevention of Cancer*: *a Global Perspective*. American Institute for Cancer Research: Washington DC

*© Cancer Research Campaign 1999*
